# Unpacking the Taxonomy of Wildland Fire Collaboratives in the United States West: Impact of Response Diversity on Social-Ecological Resilience

**DOI:** 10.1007/s00267-025-02170-w

**Published:** 2025-04-21

**Authors:** Jaishri Srinivasan, Kelly Jones, Melinda Morgan

**Affiliations:** 1 Department of Geography and Environmental Studies, University of New Mexico, Albuquerque, NM USA; 2 College of Agricultural, Consumer and Environmental Sciences, New Mexico State University, Las Cruces, NM USA; 3 Department of Geography and Environmental Studies, University of New Mexico, Bandelier East 108B, Albuquerque, NM USA

**Keywords:** Wildfire geography, American West, Collaborative governance, Social-ecological resilience

## Abstract

We offer the first study unpacking the taxonomy of collaboratives that undertake wildland fire management and how that taxonomy relates to resilience. We developed a comprehensive inventory totaling 133 collaboratives across twelve states in the western United States. We extracted each collaborative’s vision, mission, program goals, actions, and stakeholder composition. Based on this data we summarize temporal and spatial trends in collaborative formation and discuss formation drivers. Furthermore, we developed a cluster map of collaboratives based on patterns of co-occurrence of collaborative vision, mission, and goals. We identify distinct co-occurrence patterns of themes emerging from qualitative coding of collaborative missions, visions, and objectives, and define three distinct collaborative archetypes based on these. Finally, using theory-supported actions linked to basic, adaptive, and transformative social and ecological resilience, we code for presence or absence of these outcomes for each collaborative. We present the resilience outcomes by state and discuss how various collaborative typologies differentially impact levels of social and ecological resilience. Our study concludes that fire management actions for adaptive resilience such as fuels reduction, tree thinning, and revegetation are most numerous but that there is an emergent phenomenon of collaboratives engaging in transformative resilience that are mostly citizen-led networked organizations reshaping the social and ecological landscapes to include prescribed burning on a larger scale than present.

## Introduction

The occurrence, intensity and severity of wildfires globally, and in the United States in particular, has multiple responsible drivers and potential solutions, though the major compounding events are four-fold: climate change (Pausas and Keeley 2021), fire weather (Lydersen, et al. 2017), a legacy of fire suppression (DellaSala, et al. 2022) and current and projected land use change (Zhong, et al. 2021). This has resulted in increased wildfire severity across several ecoregions (Parks, Holsinger, et al. 2018), while large expanses of non-forested regions experienced a fire surplus due to introduced annual grasses and anthropogenically-induced ignitions (Parks, Miller, et al. 2015).

In the United States, this wildfire risk directly affects homes and people. In addition to homes and people, more frequent and high-intensity wildfire affects ecological integrity of headwater forests that provide drinking water to millions of people (Jones et al. 2023). The protection of source water in the western US has become a major priority for government and non-government agencies. More recently, there has been increasing concern about the role of wildfires in carbon storage, as many places in the US try to reach climate goals (Volkova, Roxburgh and Weston 2021) though a study has found that despite increased emissions North America will be a carbon sink over the 21st century (Balshi, et al. 2009). Though fire has played a prominent role in biodiversity evolution, the incidences of human activity exacerbating wildfire frequency and severity is now negatively impacting ecosystems and habitats (Kelly, et al. 2020) with cumulative effects at landscape scale being losses of closed-canopy conditions and increases in open canopy conditions (Reilly, et al. 2017). For these reasons, we have adopted a broad framing of wildfire management to include all collaborative activities that have tangible or intangible and direct or indirect impacts on wildfire mitigation or risk including but not limited to fuels reduction, forest ecosystem resilience, fire adapted communities, prescribed burning, source water protection, headwater and watershed restoration, biodiversity protection, community health, economic impacts and so on.

More than half the land in the contiguous Western states is federally owned and managed (Edwards and Sutherland 2022), and in the face of ever-increasing severity and scale of wildfires, suppression costs, largely funded by the government, escalated to $1.76 billion in 2020 (Halofsky, Peterson, and Harvey 2020). The USDA Forest Service received ~$5.5 billion in funding from the Bipartisan Infrastructure Law of 2021 to support implementation of the agency’s 2022 Wildfire Crisis Strategy from 2022–2026 (Charnley, Davis, and Schelhas 2023). Despite these investments, the economic and health impacts of wildland fires are projected to worsen, and the cost of fire suppression is expected to worsen (Burke et al. 2020) (Yung et al. 2022) (Jones et al. 2023). Scaling up mitigation often requires substantial collaborative arrangements among agencies, between agencies and the public, and across different land ownerships (Quinn-Davidson and Varner 2012), and this involves incorporating different conceptualizations and framings of wildfire risk (Essen et al. 2023). Shifting framings of wildfire risk from simple risk to complex risk require a shift to more collaborative and networked approaches to address risk across federal and private lands which involves knowledge sharing, shared power and responsibility, and incorporating local context and diverse knowledges (Essen et al. 2023).

Given the different land ownership types across the western US, wildfire management has become a multi-stakeholder collaborative endeavor. There is a large and growing understanding that community archetypes and social context differentially influence adaptive capacities (Paveglio et al. 2015) (Carroll and Paveglio 2016) (Carroll and Paveglio 2016) (Evers et al. 2019). Community-agency dynamics are increasingly important in fostering wildfire preparedness and acceptance (McCaffrey 2015), and the diversity of within-community stakeholders and interest groups influences mitigation and preparedness actions (Palsa et al. 2022) emphasizing the importance of collaborative governance approaches to wildfire response. Given the importance of collaborative governance in wildfire mitigation, it is increasingly crucial to understand the diversity of collaborative configurations existing and how this diversity results in different adaptive capacities of communities as well as how it affects landscape heterogeneity and management. The goal is to identify trends and patterns in collaborative configurations and the social and ecological outcomes they produce that will serve as a guideline for understanding how to align governance practices, program activities, and funding for different collaborative types for improved outcome effectiveness.

In this paper, we develop a unique inventory of self-organized collaborative organizations that have emerged over the past few decades in the western United States in response to the growing threat of wildfires. Collaboration involves stakeholders in a process of consensus building to address pressing environmental challenges at multiple scales and vary widely, ranging from small watershed councils to regional ecosystem collaboratives to groups addressing large-scale policy issues (Margerum 2008). There are three broad categories of collaboratives—citizen-based or grassroots groups, interagency-based groups, and cross-sector groups (Moore and Koontz 2003) (Diaz-Kope and Miller-Stevens 2015) (Diaz-Kope et al. 2015). With a diversity of collaborative types and social-ecological systems, we can also expect a diversified approach to achieving resilience outcomes. With our inventory featuring 133 collaboratives ranging from local community fire safe councils to multi-state collaborative networks and supporting organizations, we focus on three research questions:

1. *What is the distribution of wildfire collaboratives across space and time in the United States West?*
2. *What is the functional taxonomy of the collaboratives and how do they influence wildfire mitigation actions?*
3. *How do the collaborative response diversities differentially shape social-ecological resilience?*

## Collaborative Governance and Social-Ecological Resilience

The variable nature of property rights in the WUI entails a commons approach and framing because adaptive management presents a challenge to traditional government, with its reliance on bureaucratic procedures, and lengthy processes of deliberation that often exhibit a lack of flexibility, timeliness and learning required for resilience (Booher and Innes 2010) (Armitage, De Loë and Plummer 2012). Collaborative governance is increasingly being seen as possessing the necessary flexibility for faster action, with better integration of knowledge systems, better utilization of distributed resources, and enabling critical learning in close proximity with changing social-ecological systems (Plummer, Armitage and de Loë 2013) (Guerrero, et al. 2015). Social-ecological resilience is the capacity to adapt or transform in the face of change in ways that support human well-being and ecosystem integrity (Chapin et al., (2010))(Biggs, Schlüter and Schoon 2015). To understand how collaborative governance contributes to social-ecological resilience we look to theories and typologies of both in subsequent sections.

### Typologies of Landscape Collaboratives

Broader literature on collaborative partnerships reveals six common dimensions including composition, structure, scope, function, process, and outcome (Seekamp, Cerveny, and McCreary, Institutional, Individual, and Socio-Cultural Domains of Partnerships: A Typology of USDA Forest Service Recreation Partners 2011). Of these, we focus on unpacking the dimensions of *composition*, defined as the make-up and organizational diversity of partnerships, *function*, defined as what the partnership was designed to accomplish, and *process* which characterizes how partnership goals are achieved, the mechanisms by which actions are carried out, and the extent to which the purpose and missions are aligned with the characteristics of their internal members and the external environment (Seekamp, Cerveny and McCreary 2011) (Mitchell and Shortell 2000).

Bidwell and Ryan (2006) investigated the role of partnership composition on collaborative activities in Oregon watershed collaboratives. They found that agricultural landowners comprised the largest portion of membership and their findings suggest that organizational affiliations may reflect a philosophical approach rather than merely being an administrative distinction (Bidwell and Ryan 2006). Some common criteria of collaboratives include shared vision and clear goals, and environmental outcome criteria, including improved habitat, land protected from development, and land management practices (Conley and Moote 2003). (Hardy 2010) and (Koontz, et al. 2004) stress the importance of issue definition which includes biophysical impairments and strategic preservation and/or restoration activities that are a priority for watershed management, as well as collaborative outcomes which (Hardy 2010) defines as the amount and type of environmental tools and environmental and social outputs.

While collaboration may lead to more effective responses, tensions in interagency relationships as well as relationships between the government and public speak to the importance of mission alignment to ensure the efficacy of wildfire response (Fleming, McCartha and Steelman 2015) (Edgeley and Paveglio 2024). Harmonizing land-use missions associated with different agencies represents a challenge (Dombeck, Williams and Wood 2004) because some federal agencies such as United States Forest Service (USFS) and the Bureau of Land Management (BLM) have different multiple use mandates, while others such as United States Fish and Wildlife Service (USFWS) and the National Park Service (NPS) have singularly oriented missions which translate into different wildfire management and land management approaches (Fleming, McCartha and Steelman 2015). Moreover, state land agency objectives prioritize economic performance and protection of timber resources without consideration of other land use objectives, creating conflicts with federal agency land management objectives (Fleming, McCartha and Steelman 2015). Research suggests that organizational resilience in the non-profit sector can be enhanced through commitment to the mission (Witmer and Mellinger 2016) as well as constraining the network to partners with a common mission (Allende et al. 2017). Participatory decision-making includes different types of participation, including voting versus commenting, setting agendas versus choosing among pre-selected options, and having equal or differentially weighted decision-making roles among members (Lenart 2006). These reinforce the importance of composition, function, and process-related elements in collaborative partnerships in achieving resilience outcomes in wildfire management.

### Typologies of Social-Ecological Resilience

SES resilience is based on the notion of strongly coupled natural and human systems that behave in complex nonlinear ways and necessitate the adoption of complex adaptive systems science (Davidson et al. 2016). In evolutionary parlance, resilience can incorporate varying characteristics of persistence, adaptability, and transformability in varying contexts (Folke et al. 2010) (Baird et al. 2021). Maintenance of diversity and redundancy, connectedness, learning, and fostering complexity thinking are among the essential principles of resilience(Biggs, Schlüter and Schoon 2015). Ecosystems with a narrower range of functional and/or response diversity have a limited capacity to adjust to change and sustain ecosystem services than ecosystems that have a broader range (Chapin et al., (2010)). Social resilience is often mischaracterized as having the goal of functional persistence in a changing environment and separate from the processes of transitions and transformations (Pelling 2012). However, social systems that incorporate social learning measures such as learning, coping, adapting, and innovating, can increase the likelihood of shifting from a persistence state through an actively navigated transformation to a new and potentially more beneficial state (Chapin et al., (2010)). Functional and response diversity is crucial to understanding both change and stasis within a SES (Leslie and McCabe 2013).

Wildfire risk in forest ecosystems has become a complex problem because of the calculus of biophysical impacts including fuels, wildfire behavior and climate, and a coupled human-natural system approach needs to be undertaken (Fischer, et al. 2016) with a focus on social and ecological resilience by incorporating diversity and heterogeneity as principles in social and ecological systems (Steelman 2016). Accordingly, we base our assessment of social-ecological resilience to wildfires on McWethy et al. (2019)’s theory-based action framework as follows:

1. *Basic resilience*—allows and supports ecosystem recovery from wildfires and helps individuals and communities recover from fire impacts. Specific action examples include allowing fire and vegetation succession to occur in settings where exposure of valued resources is low and addressing direct (e.g., loss of infrastructure) and indirect (e.g., smoke pollution) impacts of wildfires on communities.
2. *Adaptive resilience*—Focus is on intensive fuel management and community planning to influence fire behavior and improve fire preparedness and response. Specific action examples include intensive vegetation management to reduce fire risk where human exposure to wildfire is high and changing climate and fuel conditions are moderate, forest thinning to reduce ladder fuels, coupling timber harvest, fuel reduction and prescribed fire treatments to reduce fire risk, and improve fire protection and response and reduce flammability of the built environment.
3. *Transformative resilience*—Implement a network of adaptive resilience goals across multiple communities and land ownerships, accept fire catalyzed transitions in ecosystems, and change patterns of social organization such as residential development, transportation, infrastructure, and regulations. Specific action examples include redesigning the location, character, and flammability of the built environment in landscapes with high exposure of valued resources to repeated wildfires, managing for a new fire regime where cheatgrass has become the dominant land cover, and accommodating novel regeneration pathways.

With respect to ecological resilience, additional complexities come into play. Adaptive ecological resilience has been categorized into four management options including (a) those that increase resistance to climate change or forestall undesired change, (b) those that promote resilient forested ecosystems that not only accommodate gradual climate-driven changes but tend to return to a prior condition after disturbance, (c) those that facilitate transitions of ecosystems from current to new conditions to promote successful climate response such as species dispersal and migration and changes in community composition, (d) and those that significantly change forest structure, particularly in forests that once experienced frequent, low-moderate intensity fire regimes through extensive logging and fire suppression, consequently realigning forests to present and/or future conditions quite different from the past (Stephens, Millar and Collins 2010). Prichard, et al. (2021) make the distinction between two types of forest fuel modification actions including fuels reduction, which reduces surface and canopy fuels via prescribed burning, thinning, or other mechanical treatment, and fuel rearrangement, which uses thinning or mechanical treatments without slash reduction.

All of these argue for consistent and comprehensive coordination between land managers and communities to deploy “seamless” treatments across public, private, and tribal lands (Lenart 2006) and successfully integrate community-based needs into wildfire management (Edgeley and Paveglio 2024). Therefore, collaborative governance in achieving resilience to wildfires is crucial.

## Methodology

The methodology section is divided into three parts. The first part discusses collaborative selection and inventory preparation. The second part describes descriptive data analysis that summarizes temporal, spatial, and categorical distributions. The third part describes how the functional and response diversity of collaboratives results in differential SES resilience outcomes.

### Collaborative Selection and Inventory Preparation

We developed an inventory of 133 wildfire collaboratives across the Intermountain West. Because the Intermountain West spans 12 western states and the complexity of parsing out collaboratives according to ecotone is higher than parsing collaboratives by political-administrative boundaries, we selected the latter option. The states covered include Arizona (AZ), California (CA), Colorado (CO), Idaho (ID), Montana (MT), Nevada (NV), New Mexico (NM), Oregon (OR), Texas (TX), Utah (UT), Washington (WA) and Wyoming (WY). Utah does have a Prescribed Fire Council, but there is little information on its activities, formation or structure that we can ascertain for this study, so it was excluded. There are also several cross-state collaboratives which include those featuring two or more state agencies and associated local agencies. The sources we drew from include the New Mexico Forest and Watershed Restoration Institute’s Collaborative Conservation Mapping Project (https://nmfwri.org/collaboration/the-collaborative-mapping-project/), a database of collaborative watershed organizations developed by Dr. Courtney Flint and her team at Utah State University, general web searches on wildfire and watershed collaboratives, and expert elicitation. The search is not exhaustive.

Our selection of collaboratives includes citizen-led groups such as Fire Safe Councils, agency-led groups such as the Sierra Forest Legacy, a non-profit that pursues legal challenges to Forest Service activities on public lands, and Sustainable Northwest, a company that drives and engages in collaborative management of forests in the Pacific Northwest, as well as mixed groups such as the Big Thompson Coalition and the Northern Arizona Forest Fund, all of which are in line with established stakeholder-based typologies (Moore and Koontz 2003) (Moore and Koontz 2003) (Diaz-Kope et al. 2015). It also features localized grassroots initiatives such as the Irvine Ranch Conservancy in California and the 5B Restoration Coalition in Idaho, landscape and watershed-scale interagency initiatives such as the Upper South Platte Partnership in Colorado and the Greater Santa Fe Fireshed Coalition in New Mexico, as well as cross-sector initiatives such as the Cascade Pacific Resource Conservation and Development in Oregon and the High Country Forest Collaborative in Colorado. We have not, however, included federally led initiatives such as the Joint Chiefs programs, because while these programs may be collaborative, the nature of the collaboration is consultative rather than truly collaborative. Furthermore, our study focuses on formal and informal bottom-up collaboratives that emerged rather than top-down wildfire programs.

These categories align with established typologies categorizing interagency governance, cross-sector governance, and grassroots governance (Diaz-Kope and Miller-Stevens 2015) (Diaz-Kope and Miller-Stevens 2015). It also involves networked organizations facilitating policy such as the California Wildfire & Forest Resilience Task Force and the Southwest Fire Science Consortium, as well as small councils such as the Smith River Alliance in California and the Boise Forest Council in Idaho, that fit with typologies based on action or policy work (Margerum 2008). We listed collaboratives such as California Fire Safe Councils, California Prescribed Burn Associations, and the Prescribed Burn Association of Texas as one collaborative when they are networks comprising other smaller regional and local collaboratives. Due to the large number of these smaller collaboratives and the paucity of information about them, we did not list them in our inventory, and they were not part of our count. This is one of the limitations of our study.

For each of the 133 collaboratives identified, the first author extracted the following data directly from their websites and any publicly available documents: duration, collaborative vision, mission statements and objectives, its geographic extent, formation drivers (if available), fire and forest restoration projects and activities, details of each activity including activity goals, stakeholders involved and funding sources and amounts (if available). Using this inventory, we coded data into several variables for analysis. These new variables are described in Sections 3.2 and 3.3. Since analysis of the collaboratives is based on archival information, it also forms one of the limitations of the study, as we did not reach out to any collaborative representatives to validate the information available.

### Analysis of Temporal and Spatial Distributions of Collaboratives

To look at temporal and spatial trends from the inventory, we counted the number of collaboratives formed in each of the twelve states and plotted a percent distribution graph of collaboratives prevalent statewide. We mapped the temporal distribution of wildfire mitigation collaborative formations by plotting each collaborative against the year it was formed. We then used the MTBS multi-agency database which maintains a database of wildfire events, severity, and acres burned across the United States from 1984–2021 (https://www.mtbs.gov/direct-download) (MTBS, 2023). For each of the 12 states, only incidences of acres burned by wildfire (excluding incidences of prescribed fire, wildland fire use, and unknown cases) were added for each year available. The resulting graph plots acres burned against the year the collaboratives were formed. This graph is intended to be read as a correlative trend, though there are numerous instances of specific catastrophic catalyzing events that led to wildfire collaborative creation. We plotted the state-by-state distribution of collaboratives by summing up the number of collaboratives in each state.

In this paper, we distinguish between (1) intentional events triggering government action through regulation, (2) recommendations or mandates for new group creation and funding provision, and (3) non-government actions through funding provision and group reorganization. Non-intentional events such as wildfires trigger incidental actions by either government or non-government entities through proposed or actual projects (Prokopy et al. 2014). Therefore, collaborative formation can either be a proactive measure for communities with knowledge of historical conditions and future trajectories or a reactive measure after sudden and catastrophic events. We classified the formation drivers for collaboratives that were obtained from the inventory (and group websites) for 96 of 133 collaboratives for which data were available on their websites about their formation. We coded the drivers as either “*Proactive*” in anticipation of sudden risks, gradual change, or a combination of the two, or “*Reactive*” in response to catastrophic events, socio-economic declines, mandates for creation and/or funding provision. Each of the 96 collaboratives was only categorized in one of these categories, and the total number of collaboratives that fell into these two categories was summed.

### Mission Orientations, Collaborative Clusters, and Stakeholder Variance

To develop mission maps and orientation analysis we used grounded theory which starts with an inductive logic but moves into abductive reasoning to understand emergent empirical findings and ensures that emergent categories are objective and devoid of interpretation (Charmaz 2008). Based on manual coding of vision and mission statements and program and project goals for each collaborative, we identified several recurring themes that we used as codes and then coded each collaborative against these categories. As the analysis proceeded, we found that the codes and the “families” they belong to are not necessarily mutually exclusive, and they often have indistinct boundaries (Glaser 1978) (Charmaz 2008). This coding exercise resulted in a heatmap of collaboratives.

The manual coding process began by the first author extracting key words and themes from collaborative vision, mission, and objectives and coding for their presence or absence (1/0) in each of the collaboratives. Where newer themes emerged as the collaborative list was populated, the author restarted the process of identifying whether the newer themes were present in the collaboratives already coded. This process was done in an iterative manner, until all possible themes and words were covered for and coded for their presence/absence in all collaboratives. Examples of themes include for instance “fuels reduction”, “fire adapted communities”, “fire resilient ecosystems”, and “prescribed burning”. These were grouped into a “Fire Management Philosophies” family that captures a spectrum (sometimes overlapping of fire management approaches based on differential risk perceptions). Themes such as “settlements, “wildlands”, “forest economies” and “rangeland management” were identified as belonging to a common family we named “Land Use Orientations”. Another example is “forest health/resilience”, “source water protection”, “watershed health/resilience” and “biodiversity and habitat” were grouped into a “Stewardship Orientations” family. Similarly themes such as “recreation and place”, “economic well-being” and “physical health and safety” were identified as “Community Well-Being Orientations”. We also had themes for ecologically oriented and socially oriented actions. We identified three different co-occurrence patterns among these themes leading to a categorization of three broad archetypes of collaboratives.

The occurrence of more than one code orientation within a family based on its presence in the mission, vision, and program goal statements also indicated that their occurrence for a collaborative was not mutually exclusive. For example, there is some overlap between the definitions of economic well-being and recreation and place in terms of rural identities and place-based attachments. We have attempted to clarify the boundaries between these definitions. The orientations that emerged from this process are in Table 1. The full table of definitions with both clear and ambiguous examples (with interpretation) is available in the Appendix Table S1.

**Table 1:** Orientations emerging from grounded analysis of collaborative vision, mission and goal statements

Families	Categories
Land Use	Rangeland Management
	Forest Economies
	Wildlands
	Settlements
Stewardship Orientations	Forest resilience and health
	Watershed resilience and health
	Source water protection
	Biodiversity and habitat
Ecological Actions	Preservation/protection
	Conservation
	Restoration
Social Actions	Action-oriented engagement
	Policy-oriented engagement
Fire Management Philosophies	Fuels removal
	Fire resilient ecosystems
	Fire adapted communities
	Prescribed burning
Community Well-Being Orientations	Recreation and place
	Economic well-being
	Physical health and safety

Subsequently, we used the Dendextend and Ape packages in R to create a hierarchical phylogram cluster of the collaboratives based on Euclidean distances between collaboratives from the heatmap which was converted to a presence-absence (1-0) dataset. We used a three-color clustering schematic to differentiate the collaboratives into three broad clusters. For each of these clusters, we conducted an exploratory correlational analysis between the categories in Table 1 using the Corrgram and Corrplot packages in R. The codes are available on request.

Additionally, we assessed stakeholder group variance for each of the 133 collaboratives to determine diversity in collaborative composition. In the inventory we categorized stakeholders into six categories including Federal, State, Local, Non-profit and Stewardship Organizations (including Tribal groups), Public and private agencies (including both commercial small and large businesses and city public water and energy utilities), and Other (academic institutions, citizen groups, recreational interests, centers, foundations and funding bodies and so on). The variance for each category was calculated for both within stakeholder groups and between stakeholder groups across all collaboratives within that state.

### Assessment of Social-Ecological Resilience

Building on the mission map above, we delved deeper into the detailed program actions for the collaboratives and categorized the actions taken according to the type of resilience they reflect. Using data on the specific actions that the collaboratives have engaged in based on their websites, we assessed the actions taken against the definitions and examples provided of basic, adaptive, and transformative resilience for social and ecological systems based on (McWethy et al. 2019)’s framework.

In our assessment of resilience from collaborative response diversities obtained from their specific fire mitigation actions, we make a clear distinction between adaptive and transformative ecological resilience. Actions contributing to adaptive ecological resilience included prescribed burning in conjunction with thinning and other fuels reduction measures, and the nature of the management options tends toward those that promote resilience and accommodate gradual changes. Actions that facilitate ecosystem transitions including the use of prescribed burning in a more extensive and wholesale way than merely slash reduction were classified as achieving transformative ecological resilience. Resistive actions were classified under basic ecological resilience according to (McWethy et al. 2019)’s framework. Actions significantly changing forest structure through logging and fire suppression were harder to classify because they essentially signify a degradation of ecosystem process and function, and a degradation in adaptive capacity as well as basic resilience. We, therefore, undertook our assessments based on other listed actions that the collaborative undertook that had more clarity and avoided assessments centered on this issue.

Each collaborative was counted only once against a social resilience achievement (either basic, adaptive, or transformative) and once against the corresponding ecological resilience achievement. No collaborative possessed more than one social or ecological resilience characteristic. We developed a presence-absence map of the resilience characteristics for all 133 collaboratives in this manner. To simplify the visualization of the result, we summed instances of each category occurrence of social and ecological resilience by state rather than by collaboration.

## Results

The results are organized into three sub-sections. The first sub-section discusses descriptive data analysis that summarizes temporal and spatial trends in fire collaborative formation and their formation drivers. The second sub-section discusses mission orientations, collaborative clustering outcomes, and stakeholder variance across all collaboratives. The third sub-section shows the resilience outcomes derived from collaborative actions with respect to fire management and to what extent the actions contribute to differential social and ecological resilience by state.

### Temporal and Spatial Formation Trends

**Fig. 1:**
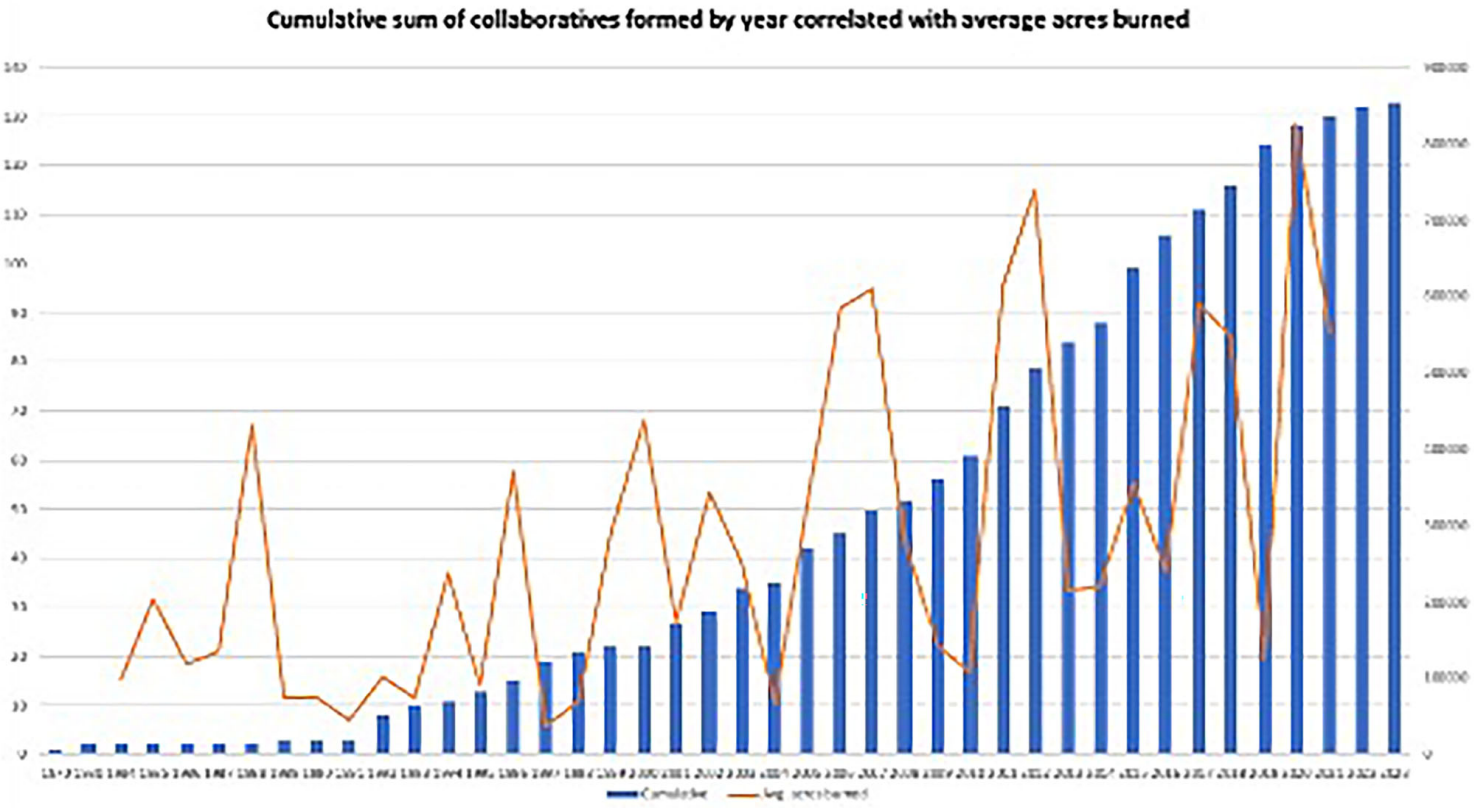
Temporal distribution of cumulative fire collaboratives correlated with annual average acreage burned across twelve states

The trends between acreage burned and the cumulative number of collaboratives formed each year imply a positive correlation between acreage burned and fire collaborative formation (Fig. 1). The average annual acreage burned trends upward since the 1980s with ever greater extremes in 2007, 2011, and 2020. There was significant collaborative formation in the period 1991–98, with higher numbers forming between 2001–05 and then again between 2011–18. The numbers then reduce from 2020 onwards. This might be a function of many factors including the pandemic which effectively slowed down collaborative action. It is difficult to say at this point whether a critical mass of collaborative formation has been reached or if there is still the potential for more collaborative formation for managing uncovered regions. Because of data availability limitations, we were not able to compare correlations between fire severity and fire collaborative formation, though we suspect it may be a more significant driver than fire extent. Table A1 in the Appendix gives the details of which collaboratives were formed in which state in which year for all 133 collaboratives.

**Fig. 2:**
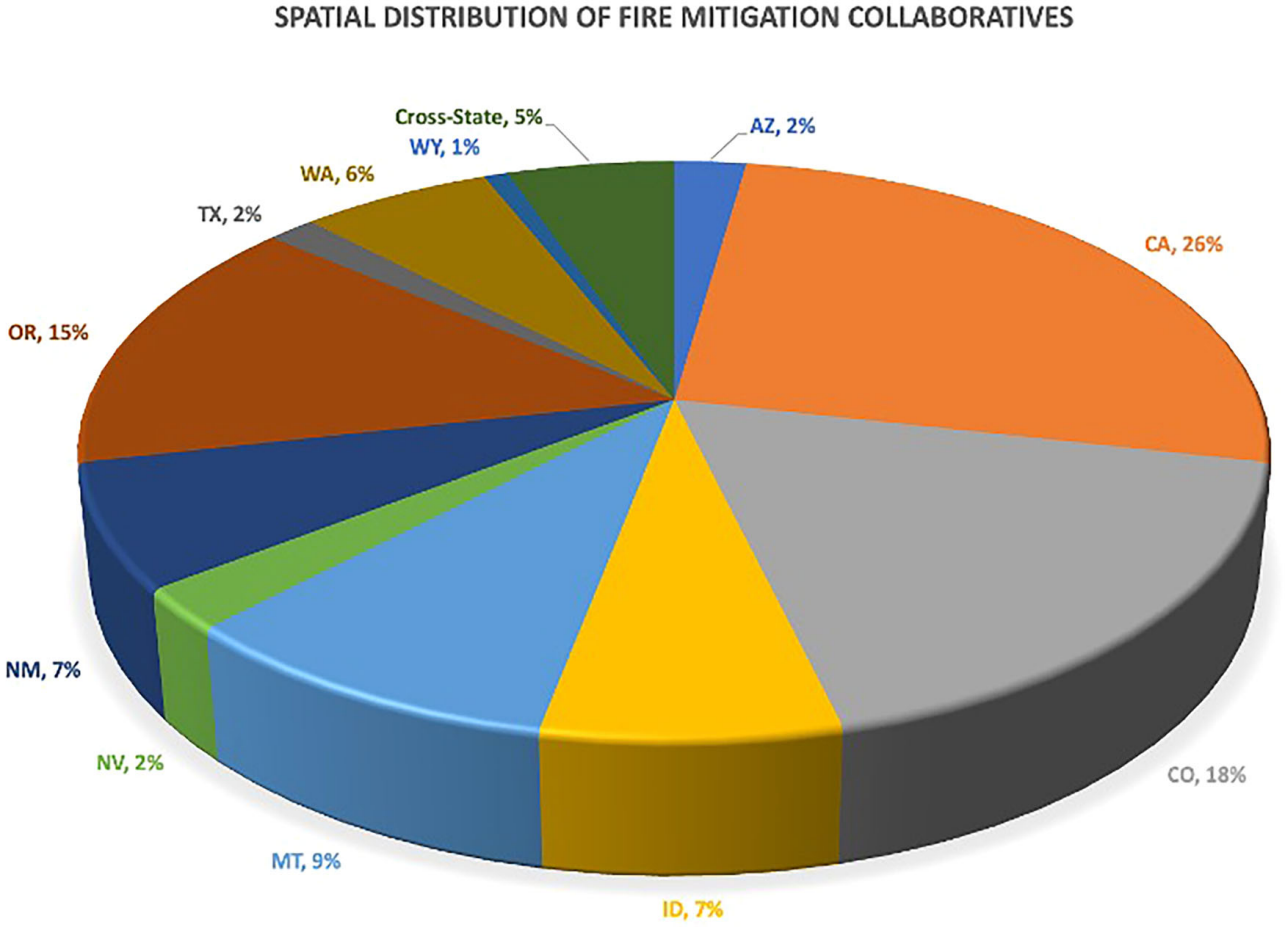
Spatial distributions of collaboratives

From Fig. 2, the first and highest number of collaboratives are observed in California (35), Colorado (24), and Oregon (20). The next highest collaborative formation is seen in Montana (12), Idaho (9), and New Mexico (9). Third order collaborative formation can be seen in Washington (8) and Cross-state collaborations (7). The lowest number of collaboratives is observed in Arizona, Texas, and Wyoming. We did not include any instance of wildfire management collaboratives in Utah in our analysis.

Out of the 96 collaboratives with information on formation drivers, the number of collaboratives proactively formed in anticipation of wildfire risk, ecological challenges, sustainable natural resource use, and community protection (N = 55) significantly outnumbered the number of collaboratives that were formed reactively in the aftermath of catastrophic events or socio-economic challenges (N = 41). The most significant reasons for proactive collective action were:

1. *Assessed risk of fire, insects, disease.* Examples include the Lake Tahoe West Restoration Partnership (CA) which identified trees and plants as being very dense near the ground creating ladder fuels and a potential crown fire risk, as well as susceptibility to insects and disease, and the Greater Rio Grande Watershed Alliance (NM) which was driven by the soil and water conservation districts with a concern for removal of invasive trees from the riparian forest and improving watershed and riparian health.
2. *Intent to resolve conflicts and reach consensus on natural resources and public lands management.* Examples include the Clearwater Basin Collaborative (ID) convened by Senator Mike Crapo following a long-running conflict regarding management of national forests, and the Whitefish Range Partnership (MT) organized with the purpose of reaching a community consensus on future management of the Whitefish range and crafting recommendations for management of the Flathead National Forest.
3. *Protecting communities, properties and values-at-risk.* Examples include the South Fork of the American River Cohesive Strategy (CA) to implement the National Cohesive Wildland Fire Management Strategy to protect the many values at risk from wildfire, and the Watershed Wildfire Protection Group (CO) which was formed to identify hazards to water supplies from wildfires and develop a watershed prioritization process.
4. *Proactive use of fire as a land management tool.* Examples include the Altar Valley Conservation Alliance (AZ) formed by ranchers motivated by a desire to return fire to the landscape and keep the valley as a working landscape, and the Prescribed Burn Alliance of Texas whose specific purpose is to promote the safe practice of prescribed burn techniques to reduce and/or eliminate fuel load build-up and preventing wildfires.

The most significant drivers of reactive formation included:

1. *Particularly catastrophic fires which were also followed in some cases by post-fire flooding and other significant ecological impacts.* Examples include the High County Forest Collaborative (CO) that formed the Forest Health Task Force in response to a mountain pine beetle outbreak, the 5B Restoration Coalition (ID) due to the Beaver Creek fire and subsequent flood events that caused significant property damage in both public and private lands, and the East Jemez Landscape Futures (NM) where continuing drought led to a mass mortality event that killed 95% of mature pinyon pine and made the landscape vulnerable to the subsequent catastrophic fires, and post-fire floods.
2. *The receipt of federal, state and/or private funds.* Examples include Alpine Biomass Collective (CA) that received a $12,000 capacity building grant from the National Forest Foundation, the South Lassen Watershed Group (CA) that was supported by a $3 million award from Cal FIRE Climate Investments Forest Health grant and Deschutes Collaborative Forest Project (OR) that was supported by Congress-established Collaborative Forest Landscape Restoration program (CFLRP) to make balanced, science-driven decisions about regional restoration projects.
3. *Reactions to economic declines or other environmental impacts that spurred collaborative discussions.* Some examples include the Amador Calaveras Consensus Group (CA) convened by the Calaveras county supervisor to discuss and come to a consensus on forest, economic and community issues following the collapse of the timber industry in the region and chronic poverty and unemployment; the Northern Sierra Partnership (CA) formed in response to protecting the ecosystems of the Northern Sierra from further commercial or residential development, and the Left Hand Watershed Center (CA) formed to oversee mine site reclamation and also driving the formation of other sister collaboratives such as the St. Vrain Partnership to protect landscapes from catastrophic wildfire risks.

### Mission Orientations, Collaborative Clustering, and Stakeholder Variance

**Fig. 3:**
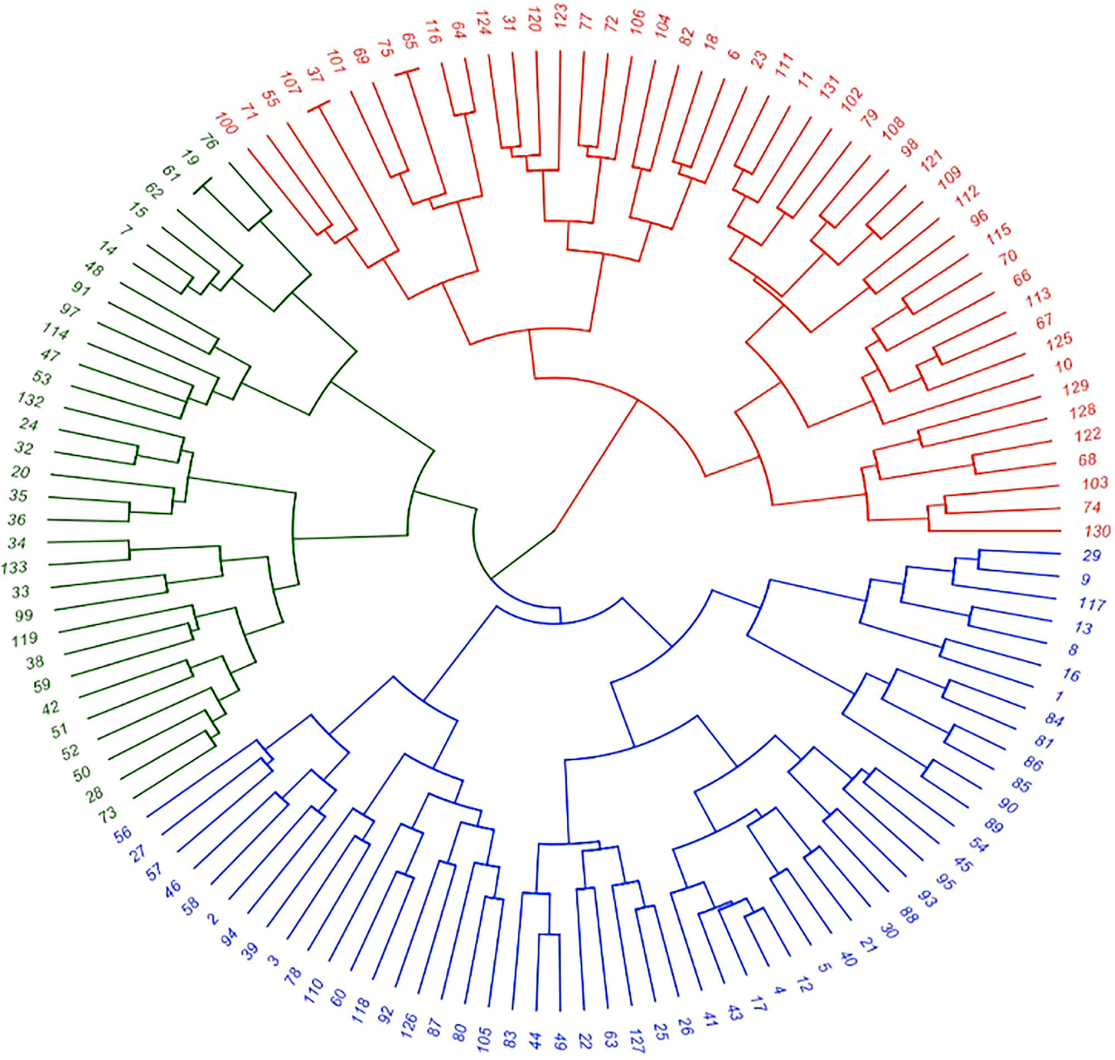
Collaboratives divided into red, blue, and green clusters. The three cluster colors showcase three distinct archetypes of mission orientation correlation patterns

Figure 3 shows the phylogram tree based on hierarchical clustering of collaboratives based on Euclidean distances and differentiated by color. We tried color differentiation for up to 6 different color clusters but settled on three colors as the main differentiating basis with distinct characteristics for each cluster. Because the collaboratives are listed alphabetically by state, the numbers in Fig. 3 correspond to the number position of the collaborative. The codes for collaborative numbers shown below can be accessed in the Supplementary Information Table S2.

**Fig. 4:**
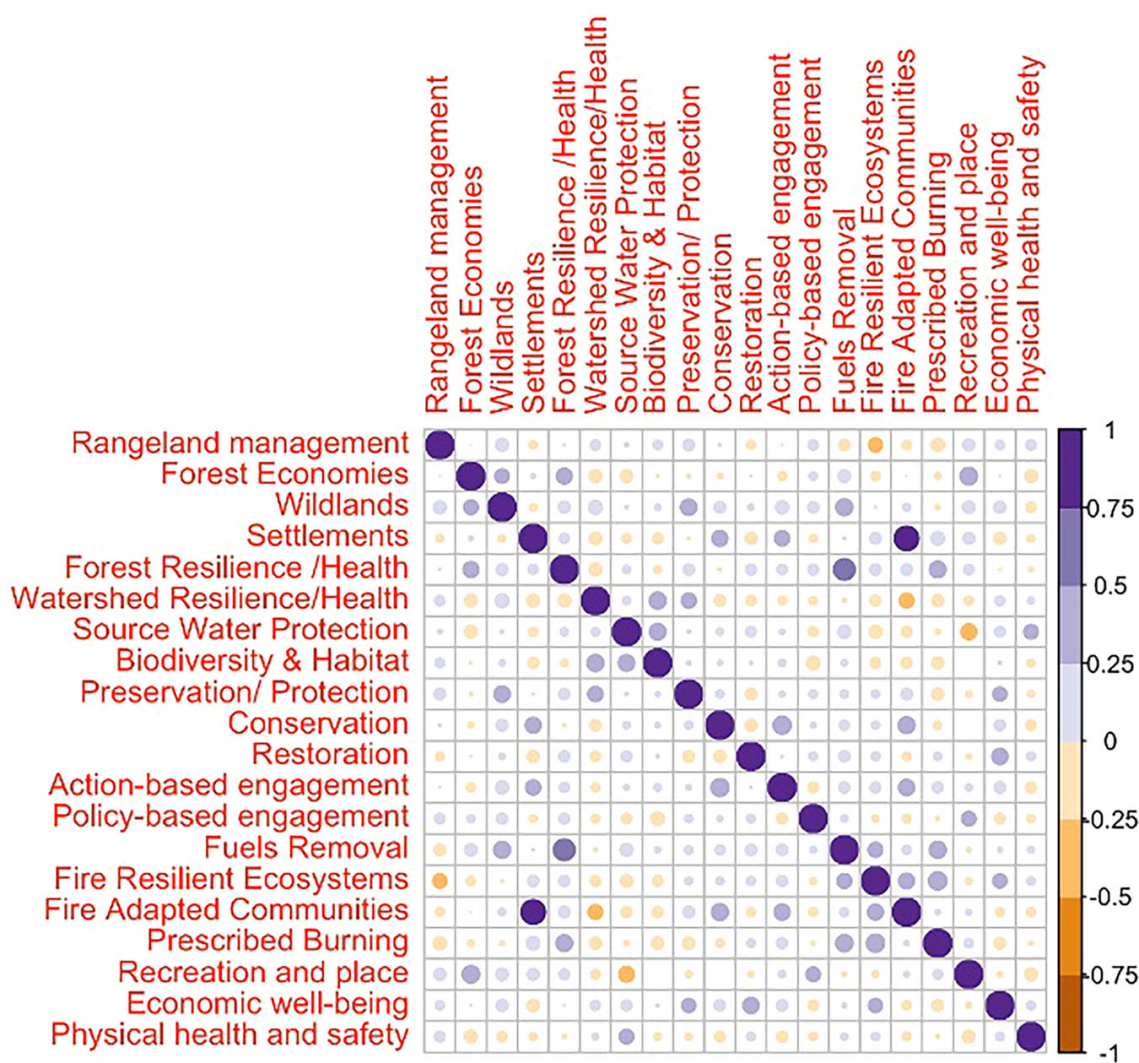
Correlation matrix for the Red cluster identified as “Core Fire Collaboratives” archetype

Figure 4 shows the Pearson correlation matrix for the Red cluster. We can see that the strongest correlation in this cluster of collaboratives is between Fire Adapted Communities—Settlements (0.75–1), followed by Fuels Removal—Forest Resilience (0.5–0.75). We use this as the defining characteristic of the Red cluster and term this cluster “*Core Fire Collaboratives*”. Furthermore, we have multiple significant positive correlations (0.25–0.5), for instance, Wildlands—Preservation/Protection, Wildlands—Fuels Removal, Forest Resilience—Forest Economies, Forest Resilience—Prescribed Burning, Prescribed Burning—Fuels Removal, Prescribed Burning—Fire Resilient Ecosystems, Settlements—Conservation, Economic Wellbeing—Preservation/Protection, Economic Wellbeing—Restoration, Biodiversity & Habitat—Watershed Resilience and Biodiversity & Habitat—Source Water Protection, among others. There are also a few instances of negative correlations (−0.25 to −0.5) including Fire Resilient Ecosystems—Rangeland Management, Recreation & Wellbeing—Source Water Protection, and Fire Adapted Communities—Watershed Resilience.

**Fig. 5:**
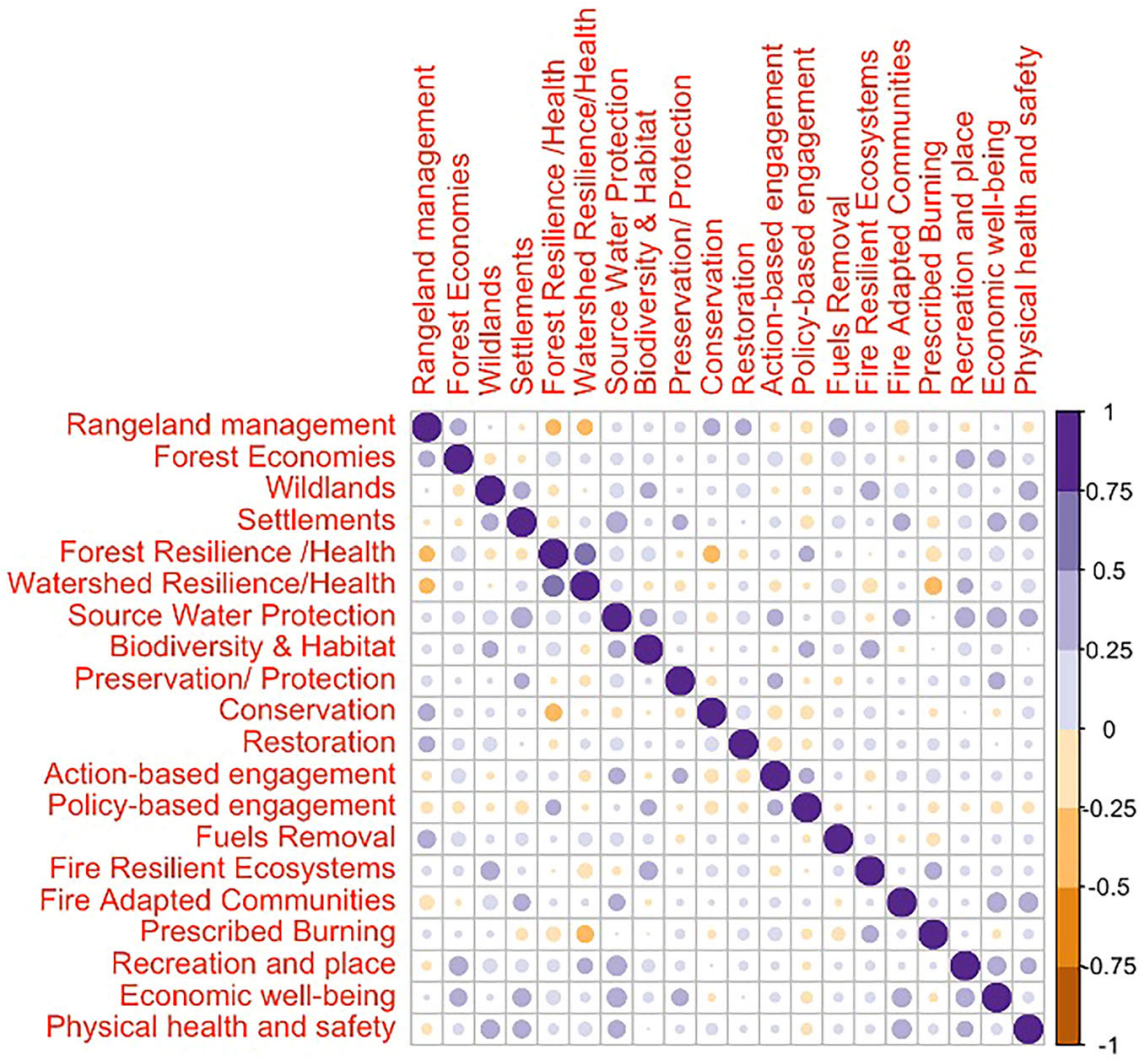
Correlation matrix for the Blue cluster identified as “Forest/Watershed Health Collaboratives” archetype

Figure 5 shows the Pearson correlation matrix for the Blue cluster. The most significant positive correlation (0.5–0.75) is between Watershed Resilience—Forest Resilience, which we take as the key characteristic of this cluster, and term this cluster as “*Forest/Watershed Health Collaboratives*”. There are multiple other positive correlations (0.25–0.5) including Forest Economies—Wildlands, Forest Resilience - Recreation and Place, Wildlands—Preservation/Protection, Wildlands—Fuels Removal, Source Water Protection—Settlements, Source Water Protection—Fire Adapted Communities, Source Water Protection—Recreation and Place, Source Water Protection—Economic well-being, Source Water Protection—Physical health and safety, Physical Health and Safety—Wildlands, Physical Health and Safety—Settlements, Physical Health and Safety—Fire Adapted Communities and Physical Health and Safety—Recreation and Place. Observable negative correlations (−0.25 to −0.5) occur with Rangeland Management—Forest Resilience, Rangeland Management—Watershed Resilience, Forest Resilience—Conservation, and Prescribed Burning—Watershed Resilience.

**Fig. 6:**
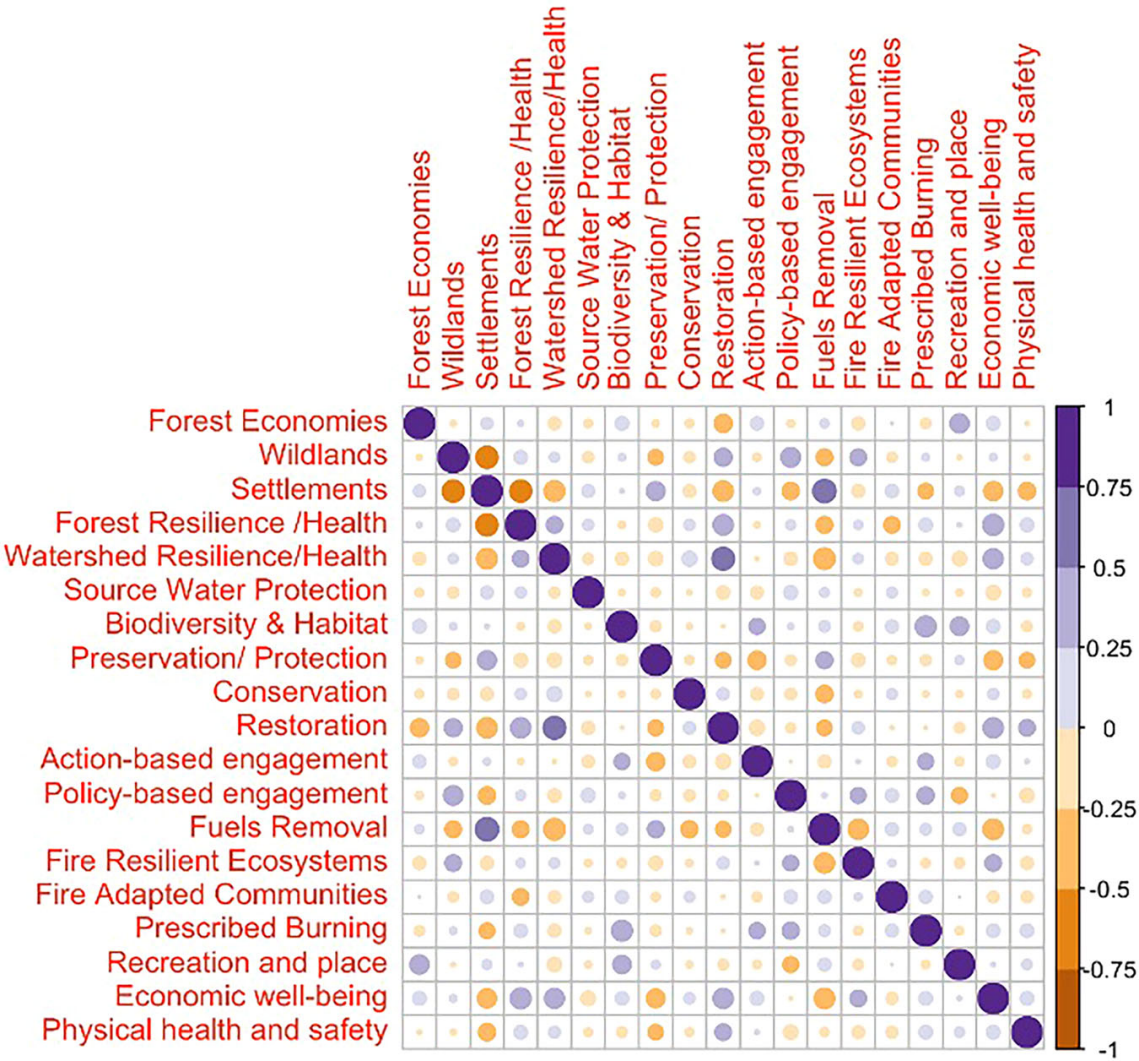
Correlation matrix for the Green cluster identified as “Mixed Use/Working Lands Collaboratives” archetype

Figure 6 shows the correlation matrix for the Green cluster. We removed the Rangeland Management variable from this assessment because the standard deviation was zero for that variable in the correlation matrix. The most significant positive correlations (0.5–0.75) occur in Fuels Removal—Settlements, and Restoration—Watershed Resilience, and the most significant negative correlations (−0.5 to −0.75) occur in Wildlands—Settlements and Settlements—Forest Resilience. We use this combination of defining negative and positive correlations to characterize the Green cluster as the “*Mixed Use/Working Land****s***” cluster. Significant observable positive correlations (0.25–0.5) occur between Wildlands—Restoration, Wildlands—Policy-based engagement, Wildlands—Fire Resilient Ecosystems, Restoration—Forest Resilience, Economic wellbeing—Forest Resilience, Economic wellbeing—Watershed Resilience, Economic wellbeing—Restoration, Prescribed Burning—Biodiversity & Habitat, and Recreation and place—Biodiversity & Habitat. Observable significant negative correlations (−0.25 to −0.5) occur between Settlements—Watershed Resilience, Restoration—Forest Economies, Restoration—Settlements, Preservation/Protection—Action-based Engagement, Fuels Removal—Watershed Resilience, Fuels Removal—Economic wellbeing, Settlements—Economic wellbeing, Settlements—Physical health and safety and Fire Resilient Ecosystems—Fuels Removal.

Table 2 shows the variance in stakeholders by state within and among collaboratives and stakeholder groups in that state. We find that the states of Arizona, Idaho, Nevada, Texas, Washington, and Wyoming had the least variance within and between stakeholder groups. Oregon and cross-state collaboratives display much higher variance, with Colorado, Montana, and New Mexico also displaying significantly higher variance, with California displaying the highest variance of all. Even discounting the fact that Arizona, Nevada, Texas, and Wyoming may have an insufficient number of collaboratives to accurately judge the implications of this variance, we find that the lowest variance is found in states that have the highest number of citizen-led collaboratives and a reduced amount of agency involvement. Collaboratives such as the Greater Flagstaff Forests Partnership in Arizona have involved citizen groups through voting on proposed fire mitigation measures. Furthermore, the rangeland management orientations in these states feature more grassroots voluntary participation of private landowners in landscape management and the proactive use of prescribed burning. Idaho and Washington too have a greater number of citizen-led collaboratives with an interest in conjoint management of public lands and forested ecosystems.

**Table 2:** Stakeholder variance within and between stakeholder groups across collaboratives by state

State	Number of collaboratives	Variance
Arizona (AZ)	3	4.77
California (CA)	35	97.97
Colorado (CO)	24	27.51
Idaho (ID)	9	4.06
Montana (MT)	12	35.34
Nevada (NV)	3	6.46
New Mexico (NM)	9	34.22
Oregon (OR)	20	16.09
Texas (TX)	2	7.72
Washington (WA)	8	6.83
Wyoming (WY)	1	1.10
Cross-State	7	20.89

Collaboratives displaying a mid-range variance (all other states excluding California) show the prevalence of agency-led and mixed-group collaborative compositions to varying extents, with higher numbers depicting more agency involvement than the lower numbers. It is interesting to note that Oregon and cross-state collaboratives do have a preponderance of centers and agencies facilitating collaborative processes, however, the nature of the facilitation tends to involve more active and democratic participation from citizen groups, NGOs, and state organizations as opposed to a more agency-skewed collaborative with fewer participants. Notable examples of these include the cross-state Southwest Fire Science Consortium and the High Desert Partnership in Oregon. The states of Colorado, Montana, and New Mexico also have more numbers of agency-led collaboratives but these feature extensive engagement with NGOs leading and facilitating initiatives in collaboration with state organizations and with the involvement of citizen groups. Therefore, the variance is higher. Examples of NGO led initiatives include the Left Hand Watershed Center in Colorado and the Zuni Mountains Collaborative in New Mexico.

California has the highest prevalence of all three types of collaboratives including agency-led collaboratives, mixed groups, and citizen-led initiatives. These include various initiatives such as the State Governor’s office-led California Wildfire & Forest Resilience Task Force, NGO led initiatives such as the North Coast Resource Partnership, the Sierra Forest Legacy, and the Watershed Center among others. There are also notable citizen-led groups such as the Yuba Forest Network and the Trinity Collaborative, and the wide disparities in stakeholder group numbers across these different types of collaboratives account for the very high variance observed among California collaboratives.

### Social-Ecological Resilience Outcomes

**Fig. 7:**
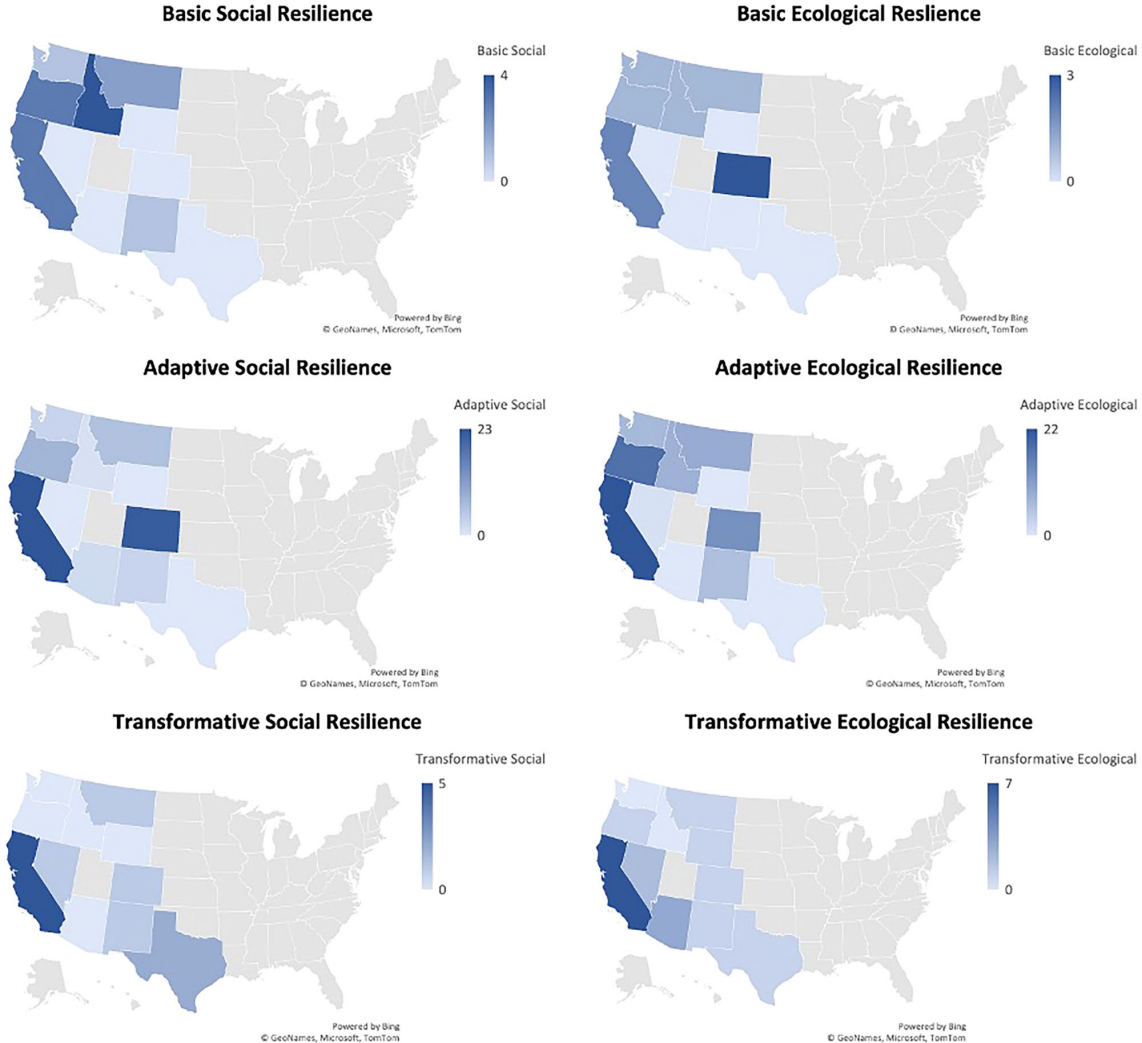
Social-ecological resilience outcomes from wildland fire collaborative action in the United States West

Figure 7 shows the resilience outcomes by state as the sum of the instances of the various resilience categories across collaboratives for each state. We start by noting that instances of basic social resilience are low throughout, though we notice 3 cases each in California and Oregon, and 4 in Idaho. Examination of these collaboratives shows that these are either very localized collaboratives such as conservancies or coalitions, or groups with a more ecological resilience focus rather than social. Conversely, basic ecological resilience, which is also low throughout shows 3 cases in Colorado which on closer examination reveal elementary levels of fuels reduction and thinning activities with restoration not being a major focus.

California had 23 incidences of achievement of adaptive social resilience through collaborative action followed by 22 in Colorado, 6 in Montana, and 8 in Oregon. Adaptive ecological resilience was achieved via 22 collaborative actions in California, 13 in Colorado, 9 in Montana, 18 in Oregon, and 7 each in Washington and for cross-state collaboratives. The correspondence between cases achieving adaptive social and adaptive ecological resilience is highest in California. Colorado, Idaho and Oregon show the most significant distance between achievement of both adaptive social and ecological resilience. Colorado collaboratives tend to implement actions fostering adaptive social resilience, whereas Oregon, followed by Idaho and Washington, are more likely to achieve adaptive ecological resilience. Transformative social resilience was observed in 5 instances in California, 4 in Idaho, and 3 in Oregon, whereas transformative ecological resilience was observed in 7 instances in California and 2 in Nevada. Collaboratives that undertake actions toward transformational resilience generally tend to achieve it both socially and ecologically, with some minor variations.

## Linking Collaborative Typologies to SES Resilience

Below we link the composition, function, and process factors (Seekamp, Cerveny and McCreary 2011) to the different types of resilience as per McWethy et al.(2019), discussing what factors of collaborative governance organized around wildland fire management contribute to basic, adaptive, and transformative resilience.

### Basic Resilience

Of the three collaboratives in California that showed basic social resilience, two showed corresponding adaptive ecological resilience, and one showed a transformative ecological resilience outcome. Of the four collaboratives in Idaho that showed basic social resilience, one showed a corresponding basic ecological resilience outcome, and the remaining three showed an adaptive ecological resilience outcome. This suggests that these collaboratives had more of an ecological focus than a social one. These collaboratives’ program actions were more likely to focus on watershed health and resilience for pre-fire management or prescribed burning. Correspondingly, of the three collaboratives in Colorado that showed a basic ecological resilience outcome, two showed an adaptive social resilience outcome, and one showed no corresponding social resilience outcome.

All collaboratives exhibiting basic ecological resilience mostly carried out fuel reduction and removal activities as basic forest management activities in addition to some instream restoration activities. The collaboratives that had basic social and ecological outcomes were smaller-scale conservancies or coalitions with missions around watershed health and resilience through stream restoration, and their actions focused on invasive vegetation species removal and vegetation monitoring activities. These actions align with established definitions of basic resilience where native vegetation recovery and successional pathways are facilitated for both basic ecosystem resilience and basic social resilience by reducing impact of fires.

### Adaptive Resilience

A significant number of cases across California, Colorado, Montana, and Oregon have corresponding adaptive social and ecological resilience outcomes. By composition, these include both volunteer community-based committees such as Fire Safe Councils in California, as well as agency-led collaborations including government-led and NGO-led partnerships, of which there is a significant number in Colorado. Fire safe councils and volunteer fire departments in California undertake activities in accordance with adaptive social resilience outcomes including improving fire protection and reducing flammability of the built environment, and represent some of the best examples of adaptive social resilience outcomes across states. Collaboratives showing adaptive social resilience outcomes show an interesting variation in their fire management philosophies. Some fire safe councils stress fuels removal activities which correlate to a settlement land use orientation with a focus on asset protection, while other collaboratives tend toward activities encouraging fire adapted communities and prescribed burning, and these tend to have more of a wildland land use orientation. In Colorado, northern Arizona, and New Mexico, we also observe that settlement land use orientations drive local and state public agencies in adaptive social resilience actions through investments in forest and watershed health and resilience for source water protection. The fire management philosophies of these collaboratives include both fuels reduction and building fire adapted communities, and community well-being orientations tend to be both economically focused as well as emphasizing physical health and safety concerns due to drinking water quality concerns.

Collaboratives showing adaptive ecological resilience vary in composition from agency-led partnerships to mixed and citizen-led collaboratives. They include interstate collaboratives in the Pacific Northwest and the Intermountain West that engage extensively in forest health and resilience activities, with fire management philosophies engaging the spectrum of fuels removal, fire resilient ecosystems, fire adapted communities, and prescribed burning. The agency-led partnerships with government and NGO participation contribute to adaptive ecological outcomes by managing public lands, the WUI intermix, and national forests to reduce fire severity and facilitate post-fire refugia. The stewardship orientations of these collaboratives tend toward a mix of forest and watershed health and resilience, and a significant focus on biodiversity and habitat protection as well. In large part, adaptive social and ecological resilience co-occur across collaboratives and represent a significant majority of collaborative activity impact.

Collaboratives in Idaho, Montana, Oregon, and Washington are also unique in that their land use orientations largely feature a mix of forest economies and wildlands, and their community well-being orientations center predominantly around economic well-being as well as recreation and place attachments. The collaboratives in these states have stakeholder groups with commercial interests in extractive industries as well as numerous organizations operating in the recreational industry. Oregon and Washington also have Native Tribes with strong interests in land stewardship in their collaborative composition. Consequently, collaborative actions in these states present some of the best examples of adaptive ecological resilience outcomes compared to other states in their attempt to balance multifunctional land use challenges and competing stakeholder interests. One case for example, the Yaak Valley Forest Council in Montana, had a preponderance of nonprofit and stewardship stakeholders aligned against the Forest Service’s proposal for a major logging project in the Kootenai National Forest that included clear-cutting significant portions of old growth and mature forest.

### Transformative Resilience

Transformative resilience outcomes are achieved by far by the smallest group of collaboratives. For the most part, transformative social and ecological resilience co-occur across collaboratives. The collaboratives that achieve these outcomes are uniquely citizen-led, demonstrating a process of emergence in grassroots level action to transform social and ecological landscapes. Notable examples of these include the California Prescribed Burn Association, the Scott River Watershed Council, the Watershed Center, the Yuba Forest Network, the Rocky Mountain Restoration Initiative in Colorado, Firesafe Montana, the Greater Santa Fe Fireshed Coalition in New Mexico, and the Prescribed Burn Association of Texas among others. These collaboratives, because of their emphasis on fire adapted communities and increased wildfire preparedness and promotion, and training of members and private landowners in conducting prescribed burns on their properties, are able to achieve transformational resilience. By composition, the collaboratives have more numbers of nonprofit and stewardship organizations.

The collaboratives engaging in transformative ecological resilience are mostly in California and Nevada with coordinated landscape management activities. These citizen-led collaboratives are reshaping the landscape through the mass coordination and implementation of prescribed burn activities. Those collaboratives that have some agency involvement such as the Watershed Center in California provide services including facilitation of burn permits for burns on private land and intensive fire practitioner training (TREX). In Nevada, the rancher-led Results Oriented Grazing for Ecological Resilience and the government-led Sagebrush Ecosystem Program both have a goal of fire-resilient ecosystem management. The Prescribed Burn Associations (PBAs) in California and Texas are nested organizations with a central organizing and coordinating committee and regional (and in some instances sub-regional) chapters with committees who coordinate with private landowners and other stakeholders to conduct prescribed burns. These citizen-driven initiatives to affect landscape-level changes show a shift in mindset from fire mitigation to actively living with fire and using it as a tool in land management.

## Discussion and Conclusion

This study maps the temporal and spatial trends of wildfire collaboratives across twelve states in the western United States and unpacks a taxonomy of collaboratives based on process and function characteristics derived from an analysis of their vision and mission statements and program goals. We also assess how the functional and response diversity of the collaboratives differentially contributes to social-ecological resilience building on emerging understandings of resilience (Selles and Rissman 2020). We find that a diversity of collaboratives formed in response to lagging top-down policy response as well as limits to federal reach on mixed land tenures. We also find that functional and process diversity of collaboratives is shaping response diversity across the landscape. There are a number of ways this occurs.

First, collaboratives that are smaller in the extent of geographic coverage such as conservancies, tend to have limited available resources and achieve basic social and ecological resilience which includes maintaining basic forest management and fuels reduction activities, and undertaking protective and preventative measures for settlement land use orientations. These are mostly locally driven with some mixed collaboratives with agency support. Cheng and Daniels (2005) found that the geographic scale of watershed planning processes influences how watershed issues are framed, with smaller-scale groups framing a direct relationship between watershed health and community wellbeing and larger-scale groups framing in terms of regional conservation efforts. We find evidence supporting this contention as larger regional groups are more likely to affect adaptive or transformational resilience because of their regional focus as opposed to basic resilience measures undertaken by smaller, more localized groups.

Secondly, we find that collaboratives that achieve adaptive resilience are not only the most numerous across states but also vary widely in stakeholder composition. The resulting differences in collaborative functional outcomes in the form of mission orientations based on land use and stewardship orientations differentially influence process outcomes including fire management philosophies and community wellbeing orientations. For example, forests in close proximity to urban areas tend toward recreational orientations while amenity destinations such as national forests may have both recreational uses as well as commodity production traditions (Seekamp, Cerveny and Barrow 2018). This creates conflicts between amenity migrants who favor preservation orientations and long-time residents who rely on forests for community economic wellbeing (Seekamp, Cerveny and Barrow 2018) with consequences for achievement of partnership synergy (McCreary et al. 2012). We find that collaboratives that functionally display a mix of forest economies and wildland land use orientations also tend to correlate in process with community economic well-being orientations as well as recreation and place preferences.

Third, transformative resilience most often emerges from grassroots-led initiatives and results in statewide networked organizations that offer various forms of tangible and intangible support for both social resilience in the form of redesigning social landscapes to reduce fire risk as well as introducing prescribed burning and other landscape management at larger scales. These coordinated efforts to reshape landscapes are few in number and most prevalent in California and Texas which are the only western states to have decentralized, networked Prescribed Burn Associations (PBAs) though they are emerging in other parts of the United States as well (Deak, et al. 2024). The main barrier is in the framing of wildfire risk as simple as opposed to complex. In simple framings of risk, technocratic governance approaches are used to achieve controllability, certainty, and security and these include wildfire severity maps, informing communities of risk, aid in prioritizing fuel treatments and suppression operations (Essen et al. 2023). Complex wildfire risk framings call for incorporation of diverse knowledges, power-sharing, site-specific strategies, and restoration of fire as a valuable component of landscape management, and such initiatives include the Fire Adapted Communities Network and Rangeland Fire Protection Associations (Essen, et al. 2023).

Our analysis of wildfire collaboratives and SES resilience point to several recommendations for enhancing both social and ecological resilience. In terms of social resilience, the main finding of our study is that transformative social resilience can be achieved by allowing emergence of citizen-led networked collaborative organizations that coordinate settlement and landscape transformation on a large scale (Cheng and Daniels 2005). But this requires studying barriers to more widespread adoption and scaling up these initiatives regionally and state policies that enable the formation and propagation of such organizations (Fleming, McCartha and Steelman 2015). Additionally, there is a need to examine collaborative efforts at multiple levels within nested organizations such as PBAs and FSCs to understand how multi-level collaboration can affect outcomes. A number of agency-led initiatives providing facilitation, science support, and prescribed fire training exchanges (TREX) in addition to other kinds of legal and policy assistance could be leveraged to greater extents to fill the gap in landscape management and fire risk reduction on varied landscape tenure regimes.

In terms of ecological resilience, first, much of wildfire mitigation action in public lands, mixed lands, and forests has involved fuels reduction including deadwood, surface, and ladder fuels removal which achieves basic resilience. In a risk framing (one in which risk is the intersection of intensity, likelihood, and susceptibility), any fuel in and of itself is a wildfire hazard though it is the intensity of the hazard which we should be concerned with. However, deadwood is an essential part of forest ecosystems and provides essential habitat for biodiversity, and decomposition is an equally essential part of forest ecosystem processes (Seibold et al. 2015). Fire practitioners need to account for this when considering mitigation options and calculating wildfire risk. Second, actions taken such as revegetation, tree thinning, aspen restoration, and conifer removal, all risk simplifying forest biodiversity to monocultures while also ignoring the impacts of climate change on forest health. Different levels of surface fuel loading present different potential intensities of hazard—discerning which levels are acceptable is exactly what collaborative governance can offer. The increasing prevalence of ghost forests points to the need to factor in considerations such as incorporating dispersal and climate migration, and succession pathways for biodiversity, and to match vegetation transitions to climate (Hill et al. 2023) which aim for adaptive and transformative resilience. Landscape and wildfire management activities that incorporate these considerations can facilitate the emergence of climate-adapted landscapes with new fire regimes.

## Supplementary information

Supplementary information

## Data Availability

Most of the data is provided within the manuscript and with the supplementary information submitted. Additional data pertaining to a sequential listing of collaboratives studied will be made available on request.
