# Supplementary material for: Unpacking the Taxonomy of Wildland Fire Collaboratives in the United States West: Impact of Response Diversity on Social-Ecological Resilience
Source: Environ Manage. 2025 Apr 21;75(6):1349–67. doi: 10.1007/s00267-025-02170-w (PMC12084270; doi:10.1007/s00267-025-02170-w)
Supplement: Supplementary file 1 — Supplementary information [file 267_2025_2170_MOESM1_ESM.pdf]

Supplementary Information

Table S1: Mission orientation definitions in detail.

Family	Category	Definition	Clear Examples	Ambiguous Examples
**Land use orientations**	Rangeland management	Refers to balancing land use between social uses of rangelands such as cattle ranching and grazing and rangeland ecosystem protection such as protection of critically endangered sage grouse and its habitat and other habitat protection initiatives. It also includes noxious weed management and removal and native vegetation planting.	Altar Valley Conservation Alliance (AZ) and its partners have indeed found common ground to restore grassland watersheds and return life to fire-adapted landscapes in order to increase native grasses, decrease brush encroachment, and make working landscapes and sustainable ranching an integral part of large-landscape conservation.<br><br>Northeastern Nevada Stewardship Group is a collaborative group based in Elko, Nevada. We address natural resource issues such as range management, stewardship of the sagebrush ecosystem, recreational opportunities, and sage-grouse recovery.	Klamath Lake Forest Health Partnership (OR) developed a strategic, accelerated restoration and priority landscape framework for Klamath and Lake Counties. This framework consolidates existing efforts within all land ownerships; identifies priority landscapes; coordinates communications; facilitates outreach; continues to promote public and landowner education; support the use of best science; and identify potential funding sources.<br><br>The Chumstick Wildfire Stewardship Coalition's (WA) highest goal is to change the social, environmental, and economic contexts in which we view fire by creating fire adapted landscapes, governments, businesses, and residents, all connected and collaborating to change the way we live and work in fire country.
	Forest economies	Refers to management of forests for timber use, developing local economies around wood production, community management of forests for timber and balancing wood products	Panhandle Forest Collaborative (ID) mission is reducing litigation, promoting sustainable operations, enhancing travel and recreation opportunities, maintaining infrastructure for timber, ranching and recreation, and conserving native ecosystems.	Big Thomson Coalition (CO) mission is to foster resilience in the watershed by providing multi-purpose and multi-stakeholder benefits to water and forest resources, as well as the wildlife and people who depend on them. Raised >$10 million since 2013 from federal, state and local funds for river

		industry with forest health and biodiversity protection.	Darrington Collaborative (WA) mission is to simultaneously create a more ecologically resilient forest, provide for increased, sustainable timber harvests on the Mount Baker-Snoqualmie National Forest near Darrington, and provide economic benefits to the community of Darrington and educational opportunities for Darrington's youth through the STEM program.	improvement projects, developed 3 large-scale river management and restoration plans, completed 10 major river enhancement projects and worked with >150 private landowners and organizations.<br><br>The California Wildfire & Forest Resilience Taskforce is a collaborative effort to align the activities of federal, state, local, public, private and tribal organizations to support programs and projects tailored to the priorities and risks of each region, and bring the best available science to forest management and community protection efforts.
	Wildlands	Refers to management of forest and/or watershed ecosystems for a variety of purposes including recreational use, local and traditional use of natural resources, balancing a multitude of different stakeholder groups social values around land use and rural identity.	North Coast Resource Partnership (CA) enhances the watersheds and communities of the North Coast region through integrated, multi-objective planning and project implementation in collaboration with North Coast partners.<br><br>The Cimarron River and Valley Watershed Coalition (NM) is a community-based organization focused on helping make the watershed a vibrant watershed flowing high quality and abundant water, healthy forests, productive agriculture, and great recreation for all to enjoy.	Northwest Community Forest Coalition (PNW) builds a broad base of support for increasing the scale and extent of community-owned forests in Washington and Oregon. Build capacity of community-based organizations to meet land acquisition needs and ensure the long-term viability of local ownership and management of community-owned forests.<br><br>High Desert Partnership (OR) brings people together to solve local problems, cooperatively. We do this by creating opportunities to listen, be heard, foster understanding, build trust, and forge unexpected relationships. By working together, we ensure our local economy, natural environment, and uniquely rural way

				of life meet the needs of today, while creating a roadmap for future generations to follow.
	Settlements	Refers to specific actions and policies around protection of public and private infrastructure and assets from fire risk. It includes protection of water treatment infrastructure for instance from wildfire risk but also predominantly protection of houses and properties from fire risk by promoting defensible space, home hardening, fuels reduction, and community engagement in fire preparedness in partnership with local agencies.	The Greater Flagstaff Forests Partnership (AZ) is a viable, respected, community-based organization that supports collaborative efforts and has been a leader in researching and demonstrating approaches to forest ecosystem restoration in the ponderosa pine forests surrounding Flagstaff, Arizona since 1996.<br><br>The mission of the Estancia Basin Watershed Health Restoration and Monitoring (NM) Project is to improve watershed health and conservation, increase water recharge, reduce property and natural resource loss from fire, and ensure consistent implementation of restoration projects through the collaborative efforts of member Soil and Water Conservation Districts (SWCD) and other stakeholders in the Estancia Basin.	The Upper South Platte Partnership (CO) is a partnership of government agencies, water providers, nonprofit organizations, and academic institutions with a common vision of sustainable and resilient landscapes, healthy forests, proactive and engaged fire-adapted communities, with safe, effective, and efficient fire response and management in the Upper South Platte watershed.<br><br>The Rivers to Ridges Partnership (OR) is dedicated to improving the quality of life for residents in the upper Willamette Valley by working together to protect and enhance the region's land and water resources and their ecosystem functions and values; and to provide environmental education and compatible outdoor recreation opportunities.
**Stewardship orientations**	Forest resilience and health	Refers to actions taken to promote, advance, restore, conserve forest health including forest management practices for improved ecosystem functioning, and sustainable community management for economic development.	The Payette Forest Coalition (ID) represents a range of interests in the Payette National Forest. Its mission is to build diverse community support for forest restoration. Landscape scale restoration can transition the forest towards improved conditions and address five goals of the Coalition's diverse interests: Wildlife, Wildfire, Watershed Health, Access and Recreation, and Restoration Economics	The Alpine Biomass Collaborative (CA) is a new collaborative group in Alpine County that believes that by improving forest and watershed health the risk of catastrophic wildfire can be reduced, and by using local labor to do this the local economy will benefit.

	Watershed resilience and health	Refers to actions taken to improve, restore, protect watershed health including soil conservation and headwater forest restoration, channel protection, streambank stabilization, instream wood placement, flow improvements, riparian restoration and protection from cattle grazing, aquatic fish stocking and conservation, water temperature regulation and so on.	South Fork American River Cohesive Strategy (CA) on a watershed scale, will work to create a fire-resilient ecosystem that supports viable populations of all native species, sustainable fisheries, functioning and restored watersheds and water quality, protected cultural resources, and diverse recreational opportunities.	The Greater Rio Grande Watershed Alliance (NM). GRGWA is a collaboration of soil and water conservation districts (SWCDs), Pueblos, agencies, and stakeholders along the Middle Rio Grande Watershed working on landscape-scale watershed restoration, with a focus on non-native phreatophyte removal from the bosque.
	Source water protection	Refers to specific activities including reforestation to improve soil health and reduce erosion, headwater forest conservation and reservoir management to improve and protect water quality for downstream drinking water consumption.	Watershed Wildfire Protection Group (CO) protects Colorado water supplies and critical infrastructure from catastrophic wildfire and other threats by maintaining healthy, resilient watersheds through collaboration, implementation, leveraging, and education.	The mission of the Cimarron Valley and River Watershed Coalition (NM) is to provide education, financial resources, communication and project implementation for improving and protecting water quality, water conservation, water use, instream flows and fisheries in the Cimarron River and its tributaries while also ensuring the long-term sustainability of the watershed's important agricultural community. The CVRWC shall operate in a collaborative manner with landowners, agencies and members of the public.
	Biodiversity and habitat	Refers to specific forest restoration practices that improve stand diversity for fire resilience, reduce the risk of high intensity fires, planting native vegetation, managing and reducing invasive vegetation, conserving habitat	The Nevada Sagebrush Ecosystem Program is a collaborative, multi-disciplinary program made up of the governor-appointed Sagebrush Council and the Sagebrush Ecosystem Technical Team, established to protect and enhance the sagebrush landscape.	The vision of the Northern Blues Forest Collaborative (PNW) is to create a vibrant Northern Blues with a restored and resilient forested landscape, strong job, recreation and business opportunities, and social harmony across the region.

		for threatened and endangered species and recreational uses.		
**Ecological actions**	Preservation/protection	Refers to preserving and protecting landscapes, watersheds, forests from use, and values-at-risk, properties, and infrastructure from risk.	The primary purpose of the Calaveras Foothills Fire Safe Council (CA) is to help protect our citizens, their property, and our natural resources from the effects of catastrophic wildland fire within Calaveras and adjacent counties.<br><br>The Clearwater Basin Collaborative (ID) enhances and protects the ecological and economic health of the forests, rivers, and communities within the Clearwater Basin by working across a diversity of interests.	The Whitefish Range Partnership (MT) mission is identifying the shared traditional livelihood and lifestyle values associated with the Whitefish Range, and working together to safeguard those values, to ensure that the exceptional natural and cultural heritage of this range may be passed on to future generations.
	Conservation	Entails conserving ecosystems and their functioning and processes.	Over the past decade, the Northern Sierra Partnership (CA) has become the booster rocket for conservation in the northern Sierra. Working together, the NSP partners have mobilized the human and financial resources to conserve over 107,000 acres across the region.<br><br>Swan Valley Connections (MT) mission is to inspire conservation and expand stewardship in the Swan Valley.	The 4Rivers Resilient Forest Collaborative (CO) mission is to envision a future where communities lead stewardship actions that promote resilient watersheds and forests on all lands within their scope, strengthening the ecological, economic, and sociocultural character of the region.
	Restoration	Entails attempts to return ecosystems and their functions, processes and biodiversity to prior or historic conditions, improving/enhancing current forest/watershed health from degraded and/or overused	The Northern Arizona Forest Fund (AZ) is a partnership between the National Forest Foundation and SRP that takes an active role in restoring forested lands and waterways on the Salt and Verde rivers watersheds. NAFF projects focus on four key areas: forest thinning and burning, stream and wetland restoration, sediment and	The 5B Restoration Coalition (ID) is working to apply the region's best thinking across sectors, developing creative and effective solutions that will generate both community and ecological resilience for future natural events.

		landscapes towards healthy ecosystem functioning.	erosion management and revegetation projects.<br><br>The Indigenous Peoples Burning Network (IMW+) is a support network among Native American communities that are revitalizing their traditional fire practices in a contemporary context.	
**Social actions**	Action-oriented engagement	Refers to proactive fostering of collaboration among stakeholders and engaging in activities to advance economic, social and ecological health, promoting science-based solutions and best practices and providing material and/or non-material support for addressing fire risk for ecological and community health. More applicable to on-ground activities and facilitation of collaboratives around those activities and education/awareness.	Started in 2018, the North Yuba Forest Partnership (CA) focuses on prioritizing, planning and executing landscape-scale forest health projects on nearly 275,000 acres of Sierra Nevada forests. Made up of diverse perspectives and interests, this collaborative will address forest health for biodiversity, habitat, clean and abundant water, carbon storage, fire protection, and recreational opportunities.	The Boulder Watershed Collective (CO) mission is to cultivate partnerships, promote community stewardships and revitalize social and ecological systems within the BWC and beyond.
	Policy-oriented engagement	Refers to the stewardship that is geared toward service provision including partnership facilitation, job creation for local economic growth, fostering social connections, undertaking legal challenges to practices that do not promote social-ecological health and resilience, provide policy recommendations to agency partners for ecosystem management. More	The Beaverhead Deerlodge Working Group (MT) works to develop common understanding among diverse stakeholders and serves as a bridge to broader constituencies on the Beaverhead-Deerlodge National Forest (BDNF). Working Group members hope to provide a way to resolve forest-level issues in a positive manner before they get to the appeal stage, resulting in more restoration work being done on the ground and in a way that achieves ecological	Southwest Fire and Climate Adaptation Partnership (IMW) is an open and inclusive group of partners with a shared vision for working together to advance fire and climate adaptation in the southwestern US.

		applicable to facilitation of collaboration around legal and policy avenues of response.	objectives as well as community benefits.	
**Fire management philosophies**	Fuels removal	Refers to actions taken to reduce fuels around communities as well as in natural and working landscapes, including logging to reduce stand density, removal of deadwood, surface and ladder fuels removal, tree thinning, invasive vegetation/weed removal, buffer space creation around infrastructure/roads and so on.	FireSafe Montana educates on the need for and how to do wildland fuel hazard identification and mitigation and the creation of survivable space.	The Fire Safe Council of San Diego County (CA) brings together community FSCs, along with local, state, and federal fire agencies in addition to other stakeholders. By working together across the county, we can utilize our combined experience, expertise, and common objectives to ensure our homes, businesses, and communities are well protected from the threats of wildfire.
	Fire resilient ecosystems	Refers to actions undertaken to improve or enhance resilience and health of forests/watersheds including managing fire regimes for healthy forest/watershed habitats and biodiversity conservation.	The Greater Santa Fe Fireshed Coalition (NM) uses a pro-active, collaborative approach to improve the health and long-term resilience of forested watersheds and communities by addressing wildfire. Their primary goal is to identify and implement high priority on-the ground projects that make the Fireshed and its communities more resilient to wildfire while maintaining and restoring resilient landscapes. This goal will be realized when fire is used as a tool for management throughout their fire adapted forests, and communities in and adjacent to these forests.	Sierra Forest Legacy's (CA) mission is to engage land managers, scientists, and stakeholders in the management of Sierra Nevada ecosystems to protect and restore the unparalleled beauty and natural values of the region.
	Fire adapted communities	Refers to advocating for the adaptation of communities to live with fire risks. Specific actions include emergency and disaster preparedness, CWPPs, ensuring safety of	The Watershed Research and Training Center (CA) is a non-profit organization located in the heart of Trinity County, California. We conduct the full gamut of land and watershed management services, lead state	The mission of the Central Texas Prescribed Fire Council is to foster cooperation among all parties with an interest or stake in prescribed fire for the purpose of promoting, conserving,

		evacuation routes, providing resources to property owners and community in mitigating fire risks on their properties and encouraging individual responsibility increasing firefighting capacity in the community, providing resources for conducting prescribed burns and smoke management in the community and so on. It is similar to asset protection, creation of defensible space, reducing fuels risk around homes/properties, also includes mentions or actions indicating fire suppression.	biomass and fire resilience partnerships, and through partnerships with communities, organizations, and public agencies we steward our landscape, create and sustain quality jobs, and connect people to the land and each other.	and expanding the responsible use of fire as a land management tool.
	Prescribed burning	Refers to proactive encouragement of the use of prescribed and/or pile/brush burning in properties, communities and publicly owned forests/watersheds.	Northern Colorado Fireshed Collaborative mission is to create resilient landscapes by facilitating an increase in the pace and scale of not only mechanical fuel reduction methods but also prescribed fires and strategically managed wildland fires across jurisdictional boundaries.	California Prescribed Burn Associations are community based, mutual aid networks that help private landowners put “good fire” back on the land. The Cal PBA website is a one-stop location for PBAs in California - providing contact information and useful prescribed fire resources.
**Community well-being orientations**	Recreation and place	Refers to well-being and values associated with recreational uses of public lands whether federal, state, local or community-owned. Can include uses of lands for hunting/fishing, tourism and biodiversity. Includes social capital built around community identification with region/place.	The Yuba Forest Network (CA) is envisioned as a central networking hub that promotes a united regional identity around the forests of the Yuba watershed and uplifts and connects the voices of active stewards of the landscape.	The Montana Forest Collaboration Network assists collaboration in forest and grassland restoration, conservation, and resource utilization for the benefit of all.

	Economic well-being	Refers to activities that promote local/regional economies including extractive activities such as mining, timber and wood products industries, cattle ranching and allied industrial and economic development activities that provide jobs/livelihoods to locals. Also refers to preserving rural values and ways of life and rural cultures around land use and local/regional identity in management of public lands.	Envision Chaffee County (CO) mission is Envisioning a thriving economy for Chaffee County and have a long-term view for growth.	Blue Forest Conservation (OR) is a mission-driven, non-profit organization, leveraging financial innovation to create sustainable investment solutions to environmental challenges.
	Physical health and safety	Refers to well-being around safety of community in the event of fire occurrence such as disaster preparedness, mitigation readiness and health protection from smoke and pollution from fire or water quality impacts.	The West Region Wildfire Council (CO) promotes wildfire preparedness, prevention and mitigation education. WRWC brings together wildland urban interface residents, fire districts, agency representatives and other partners to better prepare for wildfire.	The Amador-Calaveras Consensus Group (CA) is a community-based organization that works to create fire-safe communities, healthy forests and watersheds, and sustainable local economies.

Table S2: Alphabetically state-ordered list of collaboratives as key for Figure 3.

Number	State	Collaborative Acronym	Collaborative
**1**	**Arizona**	AVCA	Altar Valley Conservation Alliance
**2**		GFFP	Greater Flagstaff Forests Partnership
**3**		NAFF	Northern Arizona Forest Fund
**4**	**California**	ABC	Alpine Biomass Collaborative
**5**		ACCG	Amador-Calaveras Consensus Group
**6**		BCWC	Battle Creek Watershed Conservancy
**7**		CFFSC	Calaveras Foothills Fire Safe Council
**8**		CFSC	California Fire Safe Councils
**9**		CPBA	California Prescribed Burn Associations
**10**		CWFRTF	California Wildfire & Forest Resilience Task Force
**11**		ESCCRP	Eastern Sierra Climate & Communities Resilience Project
**12**		FRSC	Feather River Stewardship Coalition
**13**		Fire Forward	Fire Forward
**14**		FSCSDC	Fire Safe Council of San Diego County
**15**		FSSC	Fire Safe Sierra County
**16**		IRC	Irvine Ranch Conservancy
**17**		LTWRP	Lake Tahoe West Restoration Partnership
**18**		MKWC	Mid-Klamath Watershed Council
**19**		NCFSC	Nevada County Fire Safe Council
**20**		NCRP	North Coast Resource Partnership
**21**		NSP	Northern Sierra Partnership
**22**		NYFP	North Yuba Forest Partnership
**23**		SRRC	Salmon River Restoration Council
**24**		SRWC	Scott River Watershed Council
**25**		SFL	Sierra Forest Legacy
**26**		SRA	Smith River Alliance
**27**		SFARCS	South Fork American River Cohesive Strategy
**28**		SLWG	South Lassen Watershed Group
**29**		SSPFC	Southern Sierra Prescribed Fire Council
**30**		TCSI	Tahoe Central Sierra Initiative
**31**		TCF	The CalFauna Foundation
**32**		TSF	The Sierra Fund
**33**		Watershed Center	The Watershed Research and Training Center (Watershed Center)
**34**		TC	Trinity Collaborative
**35**		WKRP	Western Klamath Restoration Partnership
**36**		YSSFC	Yosemite Stanislaus Solutions Forest Collaborative

**37**		YFN	Yuba Forest Network
**38**		YWPFSC	Yuba Watershed Protection & Fire Safe Council
**39**	**Colorado**	4RRFC	4Rivers Resilient Forest Collaborative
**40**		ARWC	Arkansas River Watershed Collective
**41**		BTC	Big Thompson Coalition
**42**		BCF	Boulder County Fireshed
**43**		BWC	Boulder Watershed Collective
**44**		CCWFHP	Clear Creek Watershed Forest Health Partnership
**45**		CPRW	Coalition for the Poudre River Watershed
**46**		CUSP	Coalition for the Upper South Platte
**47**		DWRF	Dolores Watershed Resilient Forest Collaborative
**48**		ECC	Envision Chaffee County
**49**		EVWC	Estes Valley Watershed Coalition
**50**		FRR	Front Range Roundtable
**51**		HCFC	High Country Forest Collaborative
**52**		LHWC	Left Hand Watershed Center
**53**		MWG	Mancos Watershed Group (MWG)
**54**		NCFC	Northern Colorado Fireshed Collaborative
**55**		PPWF	Peaks to People Water Fund
**56**		RGHRP	Rio Grande Headwaters Restoration Project
**57**		RMRI	Rocky Mountain Restoration Initiative (RMRI)
**58**		SFHFHP	San Juan Headwaters Forest Health Partnership
**59**		USPP	Upper South Platte Partnership
**60**		WWPG	Watershed Wildfire Protection Group
**61**		WRWC	Western Region Wildfire Council
**62**		WAP	Wildfire Adapted Partnership
**63**	**Idaho**	5BRC	5B Restoration Coalition
**64**		BFC	Boise Forest Coalition
**65**		CBC	Clearwater Basin Collaborative
**66**		KVRI	Kootenai Valley Resource Initiative
**67**		LFRG	Lemhi Forest Restoration Group
**68**		PFC I	Panhandle Forest Collaborative
**69**		PFC II	Payette Forest Coalition
**70**		SVWFC	Sawtooth Valley Wildland Fire Collaborative
**71**		SBFHC	Shoshone Benewah Forest Health Collaborative
**72**	**Montana**	BDWG	Beaverhead Deerlodge Working Group
**73**		BC	Blackfoot Challenge
**74**		CRC	Clearwater Resource Collaborative
**75**		CGWG	Custer Gallatin Working Group
**76**		Firesafe Montana	FireSafe Montana

**77**		KFSC	Kootenai Forest Stakeholders Coalition
**78**		MFCN	Montana Forest Collaboration Network
**79**		SCC	Southwestern Crown Collaborative
**80**		SVWC	Stillwater Valley Watershed Council
**81**		SVC	Swan Valley Connections
**82**		WRP	Whitefish Range Partnership
**83**		YVFC	Yaak Valley Forest Council
**84**	**Nevada**	NNSG	Northeastern Nevada Stewardship Group
**85**		ROGER	Results Oriented Grazing for Ecological Resilience
**86**		SEP	Sagebrush Ecosystem Program
**87**	**New Mexico**	CWA	Cimarron Watershed Alliance
**88**		EJLF	East Jemez Landscape Futures
**89**		EBC	Estancia Basin Watershed Health, Restoration & Monitoring Committee
**90**		GRGWA	Greater Rio Grande Watershed Alliance
**91**		GRWUI	Greater Ruidoso Wildland Urban Interface Working Group
**92**		RGWF	Rio Grande Water Fund
**93**		SC	Sandia Collaborative
**94**		GSFFC	The Greater Santa Fe Fireshed Coalition
**95**		ZMC	Zuni Mountains Collaborative
**96**	**Oregon**	APWC	Applegate Partnership & Watershed Council
**97**		BRC	Baker Resources Coalition
**98**		BFC	Blue Forest Conservation
**99**		BMFP	Blue Mountains Forest Partners
**100**		CPRCD	Cascade Pacific Resource Conservation & Development
**101**		CSP	Clackamas Stewardship Partners
**102**		DCFP	Deschutes Collaborative Forest Project
**103**		HDP	High Desert Partnerships
**104**		JDBP	John Day Basin Partnership
**105**		KLFHP	Klamath Lake Forest Health Partnership
**106**		LSG	Lakeview Stewardship Group
**107**		LRP	Lomakatsi Restoration Project
**108**		OFRC	Ochoco Forest Restoration Collaborative
**109**		R2R	Rivers to Ridges Partnership
**110**		RFP	Rogue Forest Partners
**111**		SBRC	Southern Blues Restoration Coalition
**112**		SOFRC	Southern Oregon Forest Restoration Collaborative
**113**		SWFC	Southern Willamette Forest Collaborative
**114**		UFCG	Umatilla Forest Collaborative Group
**115**		WRCFC	Wild Rivers Coast Forest Collaborative

**116**	**Texas**	CTPFC	Central Texas Prescribed Fire Council
**117**		PBAT	Prescribed Burn Alliance of Texas
**118**	**Washington**	CSWC	Chumstick Wildfire Stewardship Coalition
**119**		DC	Darrington Collaborative
**120**		NCWFHC	North Central Washington Forest Health Collaborative
**121**		NWFC	Northeast Washington Forest Coalition
**122**		OFC	Olympic Forest Collaborative
**123**		Pinchot Partners	Pinchot Partners
**124**		SGPC	South Gifford Pinchot Collaborative
**125**		TSFC	Tapash Sustainable Forest Collaborative
**126**	**Wyoming**	WPFC	Wyoming Prescribed Fire Council
**127**	**Pacific Northwest**	NBFC	Northern Blues Forest Collaborative
**128**		NCFC	Northwest Community Forest Coalition
**129**		Sustainable NW	Sustainable Northwest
**130**	**Intermountain West**	2-3-2 CSP	2-3-2 Partnership
**131**		IPBN	Indigenous Peoples Burning Network
**132**		SFCAP	Southwest Fire and Climate Adaptation Partnership
**133**		SFSC	Southwest Fire Science Consortium
